# Supplementary material for: Prediction of Adverse Post-Infarction Left Ventricular Remodeling Using a Multivariate Regression Model
Source: Diagnostics (Basel). 2022 Mar 21;12(3):770. doi: 10.3390/diagnostics12030770 (PMC8947346; doi:10.3390/diagnostics12030770)
Supplement: Supplementary file 1 [file diagnostics-12-00770-s001.zip › diagnostics-1602318-supplementary.pdf]

diagnostics

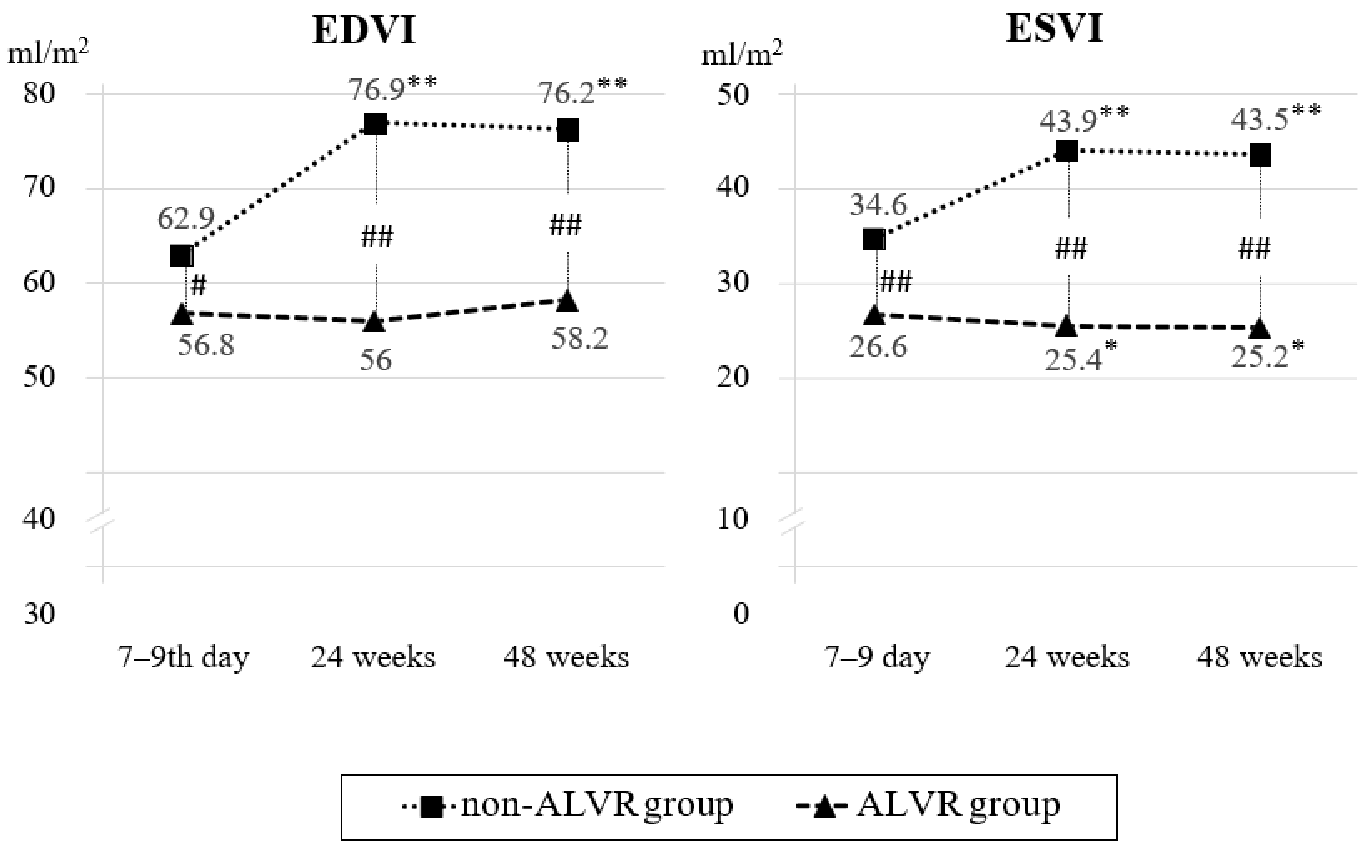

Figure S1. Dynamics of indexed values of EDV and ESV in the comparison groups. Note: * $p < 0.05$, ** $p < 0.01$— significant differences between the values on days 7–9 and subsequent visits; # $p < 0.05$, ## $p < 0.01$—significant intergroup differences; EDVI, end diastolic volume index; ESVI, end systolic volume index.
